# The GIVE Database: Acoustic and Perceptual Characteristics of Infant Emotional Vocalizations in the First Year of Life

**DOI:** 10.1111/desc.70304

**Published:** 2026-11-11

**Authors:** Maël Mauchand, Irene Lorenzini, Maya Gratier, Lucie Pugin, Andreia Gomes‐Fernandes, Leonardo Ceravolo, Didier Grandjean, Manuela Filippa

**Affiliations:** ^1^ Department of Psychology University of Geneva Geneva Switzerland; ^2^ Swiss Center for Affective Sciences University of Geneva Geneva Switzerland; ^3^ Department of Psychology University of Paris Nanterre Nanterre France; ^4^ Laboratoire Éthologie, Cognition, Développement (LECD) University Paris Nanterre Nanterre France

**Keywords:** acoustic features, affective communication, emotional valence, infant vocalizations, perceptual validation, vocal database

## Abstract

Infants communicate their affective states through a rich repertoire of preverbal vocalizations that support early social interaction and guide caregiver responsiveness. However, research on emotional infant vocalizations is limited by the lack of standardized, comprehensive, and perceptually validated datasets. To address this need, the Geneva Infant Vocalization of Emotion (GIVE) database includes 360 annotated vocalizations from infants aged 0–12 months across positive, neutral, and negative valences. Vocalizations were extracted from naturalistic parent–infant interactions, manually segmented, and annotated based on the emotional valence of the vocalization and its auditory interactional context. Acoustic features were characterized using the eGeMAPS parameter set, and a large‐scale adult perceptual validation provided ratings of valence, cuteness, and aversiveness for each sound. As an example of an individual‐level factor, we also examined how parenthood influences adults’ perception of infant vocal emotional cues. Results reveal clear perceptual distinctions across valence categories, with acoustic properties contributing to evaluations of emotional valence, cuteness, and aversiveness. Ratings of cuteness and aversiveness further differentiated vocalization categories, while age‐related variation in responses to vocalizations produced by infants of different ages, alongside differences associated with parental status, illustrates GIVE's potential for investigating developmental and experiential influences on the interpretation of infant vocalizations. A subset of 120 experimentally validated vocalizations, representative of the full set, was selected and acoustically characterized, providing a resource for research on early emotional communication, parental sensitivity, neuroimaging, and computational modeling of infant vocal behavior.

## Summary

- This paper presents a novel, perceptually and acoustically validated database of infant non‐cry emotional vocalizations across the first year of development.
- Emotional and higher‐level perceptual characteristics of infant vocalizations are reliably perceived by adults and associated with specific acoustic parameters.
- The perceived emotional expressivity of infant vocalizations progressively increases and refines after 6 months of age.
- The study suggests a cuteness schema for infant vocalizations that is partly independent from emotional valence and remains stable across development.

## 1 Introduction

Emotional vocal expression plays a pivotal role in human ontogenesis. In the first months of life, infants rely on coordinated emotional signals, combining facial and bodily movements with vocal sounds, to ensure their survival. With these signals, infants manifest psychophysiological states and needs, thus calling for the care and attention of the adults around them. These early interactions establish the foundations of attachment bonds and reveal the infant's capacity for intersubjectivity (Trevarthen 2017). Vocally, infants produce emotional signals through vocalizations whose rhythmic and melodic patterns are highly structured and embedded in communicative exchanges (Trevarthen 2008; Wermke and Mende 2009). As development progresses, anatomical maturation of the vocal tract and increasing motor control of the oral effectors further expand infants’ ability to express a richer and more nuanced range of emotions through their voice (Mugitani and Hiroya 2012). Prior studies showed that negative emotions (e.g., discomfort, distress) are typically expressed through longer, higher‐frequency cries, while positive emotions (e.g., joy, satisfaction) are conveyed by babbling and laughter; acoustic analyses reveal that these emotional vocalizations differ in duration, frequency range, and harmonic structure, and these features become more refined with age (Li et al. 2021; Mauchand et al. 2026; Papaeliou et al. 2002; Scheiner et al. 2002). The present paper has two aims: to build a systematically validated database of infant vocalizations, and to characterize how their emotional valence is perceived by adult listeners across the first year of life.

### 1.1 Adult Perception of Infant Vocal Emotions and Remaining Gaps

Given the key role of vocalization in the infants’ social world, it is crucial to understand how such vocal expressions are perceived and interpreted as emotional signals by adults. This issue is of theoretical interest, as it informs our understanding of how human infants communicate affective meaning before acquiring language, and how it integrates with and develop into “typical” adult emotion communication. The question also has significant applied implications regarding adults’ (and especially infants’ caregivers) abilities to identify, understand, and respond appropriately to an infant's states and needs (Cristia and Coffey 2024; Feldman 2007, 2012; Wass et al. 2020).

Previous studies have mainly focused on infants’ perception of emotions and valences, but fewer efforts have been directed to exploring how adults understand infants’ emotional expressions. In one such study (Galati and Lavelli 1997), authors found that adults showed higher agreement when interpreting the valence of facial expressions in older compared to younger infants. Research on how adults interpret infants’ emotional vocal expressions has focused predominantly on infant cries, while far less attention has been devoted to other preverbal vocalizations such as laughter, babbling, and protophones. Cries have received the greatest empirical interest due to their evolutionary relevance as salient distress signals and their strong impact on adult attention and caregiving behavior (Wang et al. 2025). Neuroimaging and behavioral studies consistently show that cries trigger robust emotional, physiological, and neural responses in adults, engaging brain systems related to attention, salience processing, and caregiving motivation (Dudek et al. 2016; Witteman et al. 2019; Young et al. 2015). Furthermore, individual differences such as parental status, gender, and attachment representations modulate neural and emotional reactivity, with parents and women generally showing heightened sensitivity to infant distress (Bornstein et al. 2017; Parsons et al. 2017). Adults rely on acoustic cues, including pitch, duration, and nonlinear acoustic phenomena, to assess the intensity and meaning of cries, leading to high levels of agreement across listeners (Corvin et al. 2025; Yoo et al. 2019). In contrast, research on adult interpretation of non‐cry vocalizations is limited. Only a handful of studies have compared responses to cries versus positive or neutral sounds, demonstrating that cries are perceived as more arousing, aversive, and cognitively distracting than laughter or speech‐like vocalizations (Dudek et al. 2016; Bornstein et al. 2017; Yoo et al. 2019). When only focusing on non‐cry vocalizations, adult listeners (and especially parents) seem to reliably determine emotional valence (Lindová et al. 2015), but more fine‐grained perception and developmental trajectories remain to be clearly assessed.

### 1.2 Cuteness Perception in Infant Vocalizations

The perception of infants by adults often involves an attribution of cuteness to their appearance and expressive cues. Research on visual cues has identified robust “baby schema” effects, whereby facial features such as large eyes, round cheeks, and small chins reliably elicit positive affect, heightened attention, and strong caregiving motivation in both adults and children (Glocker et al. 2009; Lorenz 1943; Zhou et al. 2022). These findings underscore the dominant role of facial information in shaping caregiving responses and in driving perceptions of likability, health, and adoptability, triggering brain activity tied to pleasure and empathy, propelling caregiving behaviors (Kringelbach et al. 2016). In contrast, the attribution of cuteness to infant vocalizations remains largely unexplored; the possibility that auditory cues may also trigger cuteness‐related responses has only recently begun to receive empirical attention. Emerging evidence suggests that adults implicitly associate positive valence with infant vocal sounds, pointing to a potential form of “auditory cuteness” (Arteche et al. 2023; Kringelbach et al. 2016). Yet, little is known about the acoustic characteristics, perceptual mechanisms, or neural correlates underlying this response, and the field remains far less developed than research on facial cuteness. Cuteness is nonetheless a dimension that a vocalization resource should capture, because it need not coincide with emotional valence: valence concerns the affective state attributed to the infant, whereas cuteness (and its assumed counterpart, aversiveness) concerns the motivational pull of the sound on the listener and is directly relevant to caregiving responses (Kringelbach et al. 2016). A database intended to support research on early emotional communication should therefore document not only how the valence of infant vocalizations is perceived, but also how cute or aversive they sound, how these evaluations relate to perceived valence, and how they change across the first year.

### 1.3 The Methodological Need for a Standardized, Validated Corpus

Despite substantial interest in infant emotional communication, research on preverbal vocalizations remains fragmented and constrained by several methodological limitations. Terminological inconsistency is a major source of fragmentation: studies employ diverse classification schemes—distinguishing cries, laughs, babbles, protophones, squeals, or growls—which complicates comparisons across findings (Marschik et al. 2022; Scheiner et al. 2002; Yoo et al. 2024). Moreover, much of the literature focuses narrowly on cries, leaving the broader repertoire of preverbal sounds understudied and limiting our understanding of their communicative functions (Long et al. 2020), with few exceptions that also include non‐cry vocalizations (e.g., Lindová et al. 2015). Methodologically, studies often rely on small, homogeneous samples that restrict generalizability (Long et al. 2020; Salvadori et al. 2022) and on brief, structured laboratory recordings that fail to capture the variability of naturalistic vocal behavior. By contrast, day‐long home recordings, still rarer but increasingly popular, offer richer data and higher ecological validity (Cristia and Coffey 2024; Yoo et al. 2024). Manual coding—still the dominant approach—is labor‐intensive, subjective, and may prove inconsistent across studies, limiting replicability (Marschik et al. 2022; Salvadori et al. 2022). Furthermore, the adoption of advanced computational and machine‐learning techniques remains limited, despite their potential to provide more objective and scalable acoustic analyses (Li et al. 2021; Pokorny et al. 2020). Acoustic feature extraction itself lacks standardization, with studies often focusing on low‐level descriptors that may capture only part of the emotional signal (Marschik et al. 2022; Li et al. 2021; Pokorny et al. 2020). Integrating perceptual data introduces additional challenges: relatively few datasets combine detailed acoustic analyses with systematic adult perceptual evaluations, and even fewer consider individual listener differences such as parental experience. For instance, Yoo et al. (2019) showed that while listeners generally agree on distress ratings, they differ in the acoustic cues they rely on, and these differences are influenced by prior exposure to infant vocalizations. Large‐scale cross‐cultural studies have begun to examine perceptual sensitivity (Cox et al. 2022; Hilton et al. 2022), yet they rarely assess how listener characteristics modulate perception. Overall, the field would benefit from larger, more diverse, and longitudinal datasets that jointly integrate acoustic features, perceptual judgments, and listener‐level variables, including parenthood, to more fully capture the complexity of preverbal emotional communication.

### 1.4 The GIVE Database and Aims of the Present Paper

The primary aim of the present study was to develop a perceptually grounded database of infant vocalizations spanning the first year of life, suitable for future experimental research on early emotional communication: the Geneva Infant Vocalizations of Emotions (GIVE) database. Few existing datasets combine acoustic characterization with systematic adult perceptual evaluation, and fewer still consider listener‐level variables. This gap motivates the present resource, and three analytic strands follow directly from it: how the valence of infant vocalizations is perceived across development, how cuteness and aversiveness constitute a second evaluative dimension, and how parental status, as a worked example of a listener‐level moderator, shapes these judgements.

To achieve this goal, we first combined naturalistic recordings of infant vocalizations with expert segmentation and pre‐categorization according to emotional valence, and conducted a large‐scale perceptual evaluation by adult listeners. This allowed the creation of a full‐fledged, annotated set of 360 vocalizations, along with an experimentally validated subset of 120 prototypical vocalizations that were perceptually validated and acoustically representative. Further analyses aimed to characterize the perception of infant vocalizations across representations of emotional valence, cuteness, and aversiveness. More specifically, they examine how these perceptual evaluations relate to the acoustic properties of the vocalizations and vary according to the age of the infants who produced them and listeners’ parental status. These analyses were intended to establish GIVE as a systematically validated resource for research on early emotional communication and adult responses to infant affective signaling.

## 2 Database Construction and Validation

The present database was constructed through a perceptually informed stimulus selection procedure combining the extraction of vocalizations from naturalistic recordings, segmentation and expert valence annotation, acoustic characterization using Principal Component Analysis (PCA)‐derived components, and perceptual evaluation by adult listeners, informing the selection of a consistent stimulus subset. A total of 360 stimuli (i.e., infant vocalizations) depicting negative, neutral, and positive infant emotions across their first year of life were extracted from everyday‐life recordings. The stimulus selection process described below is also presented in a previous article focused on acoustic features of the same stimuli (Mauchand et al. 2026).

### 2.1 Recording

The vocalizations were extracted from two corpora of babies’ recordings in natural environments. Both corpora were created using relatively comparable recording methods. Subjects were mostly biparental, monolingual French families in Paris, and recordings were made during regular days without specific prompts, using portable recording devices placed as close to the infant's mouth as possible without disturbance. Parents were aware of the recorder but were asked to behave normally as if it wasn't there.

The first corpus was composed of 1953 audio recordings lasting on average 15 min each. They were collected longitudinally, once a month, from 24 monolingual francophone infants, aged between 1 and 5 months. All recordings were made in naturalistic context, at home or in a lab, with a digital audio recorder (Korg Sound on Sound Unlimited Track Recorder) placed near the infant while they interacted normally with their parents. From these recordings, short segments containing sound produced by infants only, with no overlap with another voice or external sound, were isolated.

The second corpus was composed of 126 audio recordings lasting approximately 30 min. They were collected cross‐sectionally from monolingual francophone infants aged 7 to 8 months (group 1, *N* = 24) and 10 to 11 months (group 2, *N* = 18). Recordings were made using the LENA recording device (a small recording device that is worn by the infant in a specially designed vest, Canault et al. 2016). The participants’ vocal productions were continuously recorded during 10 h on average within a regular, at‐home day spent with at least one of the habitual caregivers. Three 30‐min segments were retained per participant, corresponding to the three continuous segments with the highest number of vocalizations as detected automatically by LENA software analysis (because LENA detects based on areas of acoustic energy, most segments contained primarily vocalizations made by the infant, closest to the device) and with guaranteed good sound quality for at least 20 of their 30 min. All families signed written opt‐out informed consent, and the procedure was approved by the CER of the Paris Cité University. For this corpus, the consent forms specified that the recordings would be listened to and analyzed anonymously with the sole aim of coding and studying infant babbling, and that they would remain strictly confidential and accessible only to the scientific personnel directly involved in the project; the raw recordings are therefore not publicly shared.

The recorders used for both corpora were comparable in recording specs (16‐bit, 16 kHz  .wav output). Both LENA and Korg Sound on Sound are portable devices, though the lighter weight and size of LENA makes it a wearable device. Nevertheless, recorders were placed at comparable distances from the infant's mouths (attached to their shirt for LENA, or in their crib/on the floor near them) and in very similar contexts, limiting device artifactual, device‐dependent differences in the analyses. Stimuli segmentation, selection, post‐processing, and feature extraction (see below) also ensured homogeneous signal quality across data.

### 2.2 Segmentation and Annotation

Recordings from both corpora were then precisely segmented into single vocalizations using Praat software. Vocalizations of interest were defined as high‐quality voiced productions from the baby, without background noise or artefacts, and lasting at least 700 ms and at most 2 s. This duration window was justified by the need of relatively homogeneous length stimuli for future experimental use requiring stimulus regularity (e.g., fMRI studies). In addition, removing long vocalizations allowed the restriction of stimuli to non‐cry (and non‐laughter), as cries tend to be much longer in duration. Non‐continuous, consecutive voiced segments within this time window were considered as part of the same vocalization if they were separated by less than 300 ms. During the segmentation process, experimenters aimed to capture as much emotional variability from the babies, by avoiding to segment vocalizations that were too similar (e.g., when the baby repeats the same vocalization) and by selecting excerpts across the whole recording time available.

Vocalizations were clustered within four age ranges to simplify analysis, based on known critical periods of vocal development: 0–3 months, 3–6 months, 6–9 months, 9–12 months. Most selected utterances were from babies in the center of a range (1–2 months, 4–5 months, 7–8 months, and 10–11 months). During the segmentation, the following information was extracted from the vocalizations: age and age range (0–3 months, 3–6 months, 6–9 months, 9–12 months), sex of the baby, duration of the vocalization, and estimated valence of the vocalization (Positive, Neutral, or Negative). This estimation was based on the qualitative evaluation of the vocalization within its auditory context (e.g., parent's speech) by the experimenters. Coding was entirely auditory: both corpora are audio‐only (a Korg digital audio recorder placed near the infant for the first corpus, and the LENA device worn in a vest for the second), no synchronized video was available at any stage, and experimenters therefore had no access to the infant's facial or bodily affect. The pre‐categorization should thus be understood as a contextually grounded expert judgement, based on the vocalization and its surrounding acoustic context, rather than as a measure of the infant's internal emotional state. Vocalizations were qualified as negative when preceded by a negative event (e.g., parent scold, loud noise) or prompting a comfort response from the parent, or preceding/following a crying phase; positive when elicited during play (either interactive play or self‐directed play, when clearly recognizable from audio) or accompanied by positive speech from the parent; neutral when elicited in the absence of a parent or stimulation. This categorization was performed by two trained master's students supervised by an expert researcher who reviewed and validated each annotation. In cases of disagreement, the vocalization was discarded. Details of each vocalization are available in the shared data repository (see Data Availability statement).

Vocalizations were retained to create a harmonized database as follows: 90 vocalizations per age range with 30 positive, 30 neutral, and 30 negative vocalizations in each range, balanced sex across age ranges, and similar duration statistics that remained representative of the original set across age range (*M* = 1.2s, SD = 0.3s). To account for volume variability across the recordings, all the selected stimuli were normalized to a mean sound pressure of 70 dB using Praat software (Boersma and van Heuven 2001). In total, 360 vocalizations (4 age ranges × 3 valences × 30 stimuli) were selected. Table S1 details the number of vocalizations and infants at each stage: 695 vocalizations (from 36 infants) were isolated and segmented from the two corpora, from which the 360 vocalizations (from 35 infants) were selected to obtain a set balanced across valences and age ranges, with harmonized but representative duration statistics. The perceptual validation described below then identified an experimentally validated core set of 120 vocalizations (from 30 infants) with perceptually consistent valences (infants contributing to two age ranges are counted once).

### 2.3 Perceptual Validation

The selected vocalizations were then used as stimuli in a perceptual experiment, in which participants were asked to evaluate several emotional aspects of the vocalizations. The aim of this experiment was first to assess the quality of the selected database for future usage and to define an experimentally validated subset with most consistent perceptual evaluations. As described in the analyses further below, it also aimed to provide some initial statistics on the emotional perception of babies’ vocalizations across the first year of life, and

#### 2.3.1 Participants

79 participants (53 women; age: *M* = 31.09, *SD* = 9.43) were recruited, either as voluntary participants through snowball sampling or for university credits via the student recruitment platform SONAsystems of the University of Geneva.

#### 2.3.2 Stimuli

Stimuli were the 360 selected vocalizations. To limit the duration of the experiment, participants were divided into three groups. Each group was attributed 112 unique vocalizations (not given to the other groups). In addition, 24 vocalizations (6 from each age range) were given to all three groups. Hence, each participant listened to 136 vocalizations.

#### 2.3.3 Procedure

The experiment was run online using Psychopy (Peirce et al. 2019) and its corresponding platform Pavlovia. Prior to the beginning of the experiment, participants completed a Huggins Pitch test to confirm that they were using high‐quality headphones (Milne et al. 2021). This test consists of the detection of a dichotically reconstructed pitch in white noise. The pitch is inaudible when using simple speakers but relatively easy to detect with good headphones; this allowed control of a good sound environment and of the attention and correct hearing of the listeners, even when completing the task at home.

Each participant completed 136 trials, each trial corresponding to the evaluation of a vocalization. In a trial, participants were first presented with a vocalization stimulus. They were then prompted with two consecutive questions. The first question asked them to evaluate the emotional valence of the stimulus (“Quelle est la valence émotionnelle de la vocalise?”—“What is the emotional valence of the vocalization?”). The question was shown together with three slider bars: Positive, Neutre, Négative (Positive, Neutral, Negative). The participant could adjust any of the three sliders depending on how much the vocalization corresponded to that valence. The choice of three sliders rather than a single, Negative‐to‐Positive slider was motivated by the more fine‐grained evaluation it allowed for participants. It also permitted a better assessment of “confusing” vocalizations which may not be well captured by a single continuum. After the valence question, they were prompted to evaluate how they perceived the vocalizations (“Comment avez‐vous trouvé cette vocalise?” “How did you find this vocalization?”), using two sliders: Mignon, Irritant (Cute, Irritating). For both questions, participants were allowed to replay the vocalization once by pressing a replay button. They could also answer “Je ne sais pas” (“I don't know”) instead of using the sliders. The proportion of “I don't know” responses was explored via mixed‐effect logistic regressions (see Results), but for all other analyses, these trials were discarded (275 Valence trials and 725 Cuteness trials). In any case, a response was required to move on to the next question/trial. In total, the experiment lasted approximately 40 min.

### 2.4 Selection of an Experimentally Validated Subset

For future research purposes, a subset of 120 prototypical vocalizations was selected based on the concordance between experimental pre‐categorization and perceptual judgements. For each vocalization, the average score of each perceptual scale was computed. A vocalization was considered “correct” if the valence scale (Negative, Neutral, or Positive) with the highest average score was the same as the experimenters’ pre‐categorization. As shown in Table 1, listeners were mostly in concordance with the experts’ pre‐classification, except for neutral vocalizations, which were often miscategorized as positive in the 6–9 months range (*n* = 15) and the 9–12 months range (*n* = 19). Of note, most “incorrect” categorizations occurred to or from the neutral scale, with very little between positive and negative stimuli.

**TABLE 1:** Classification of vocalizations based on their highest‐scoring valence scale. The bolded values represent the number of “correct” vocalizations, i.e., those whose highest‐scoring valence corresponded to their pre‐categorization.

		Number of vocalizations perceived as:		
Age Range	Pre‐categorized valence	Mostly negative	Mostly neutral	Mostly positive
0–3 months	Negative	**23**	6	1
	Neutral	1	**22**	7
	Positive	1	11	**18**
3–6 months	Negative	**16**	13	1
	Neutral	2	**18**	10
	Positive	3	6	**21**
6–9 months	Negative	**23**	6	1
	Neutral	2	**13**	15
	Positive	2	0	**28**
9–12 months	Negative	**28**	2	0
	Neutral	1	**10**	19
	Positive	2	1	**27**

The validated subset was created by selecting, for each age range and each valence, the 10 “correct” utterances which scored highest on their appropriate valence scale. This resulted in 4 Age Ranges × 3 valences × 10 vocalizations = 120 stimuli. As shown in Figure 1, this selection retained the trends reported in the analyses, while maximizing the appropriate valence scores and minimizing noise in the other scores.

**FIGURE 1:**
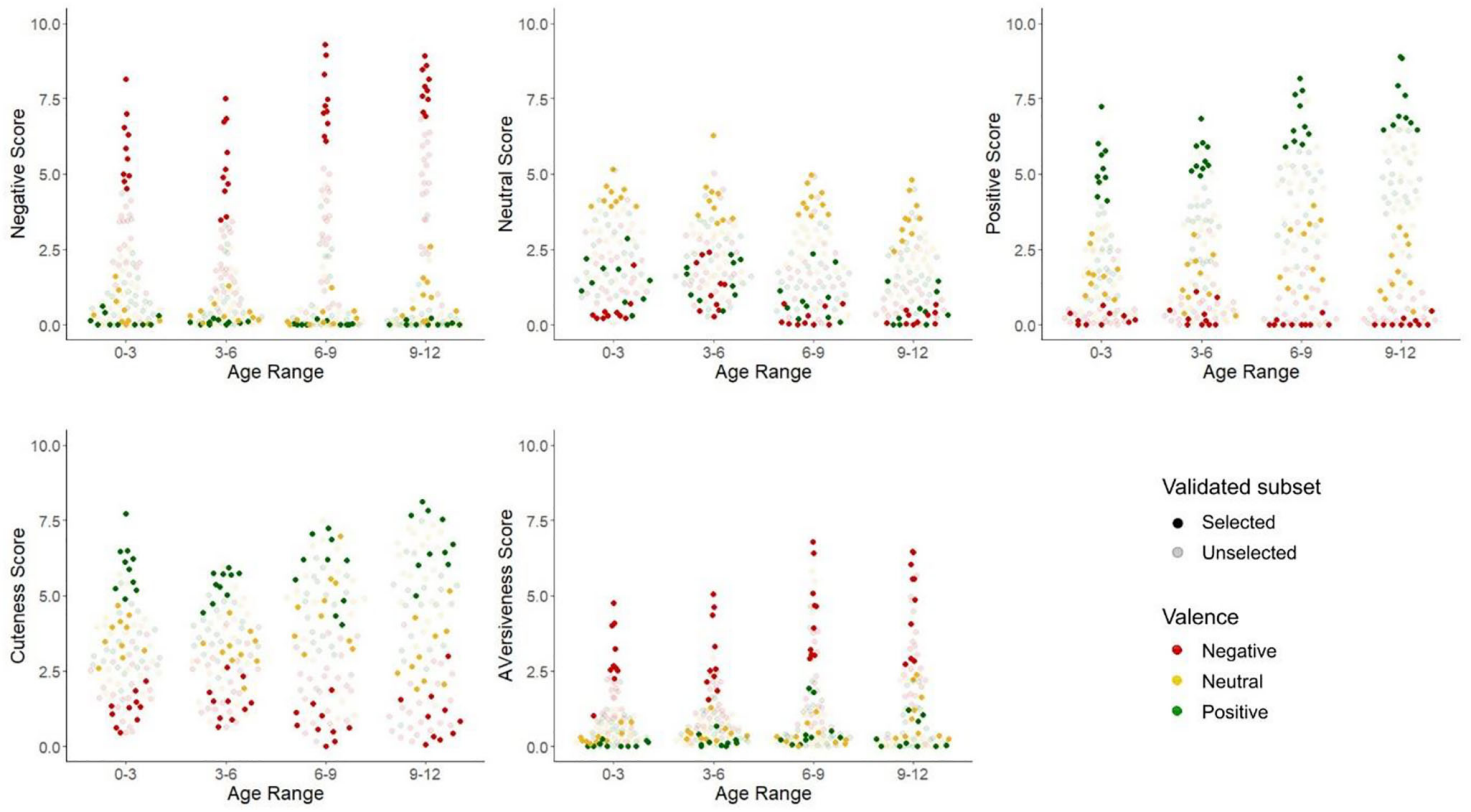
Predicted perceptual responses for each valence type at each age range. Each panel represents scores from a given scale (top: Negative/Neutral/Positive, bottom: Cuteness/Aversiveness). Dots represent the predicted response from the model, and error bars are the model's 95% confidence intervals.

Inter‐listener agreement in the subset was calculated via one‐way random intraclass correlation coefficients for each valence score. ICCs suggested moderate to good reliability in positive (ICC = 0.53) and negative (ICC = 0.73) evaluations within the subset, but relatively poor reliability in the neutral evaluations (ICC = 0.31). Still, reliability was greatly improved when compared to ICCs computed from the entire dataset (positive: ICC = 0.40, negative: ICC = 0.57, neutral: ICC = 0.17).

## 3 Analyses

### 3.1 Acoustic Characterization

Acoustic parameters were extracted using the extended Geneva Minimalistic Acoustic Parameter Set (eGeMAPS), (Eyben et al. 2016) in openSMILE software (Eyben et al. 2010). The eGeMAPS is a set of 88 acoustic parameters related to the frequency, loudness, spectrum, and rhythm, which are relevant to emotional speech analysis. The parameters were extracted dynamically across sound samples, and then passed through mathematical functions (e.g., mean, standard deviation, maximum) applied to the whole duration of the vocalization. To reduce these 88 parameters to a restricted number of interpretable values, a PCA with varimax rotation was performed on the database, resulting in 5 rotated components (RC). Analysis of the PCA scores is described in detail in Mauchand et al. (2026). In summary, the five components isolated five families of acoustic characteristics of the stimuli (see Table 2):

- RC1: Spectral energy and pitch
- RC2: Loudness and resonance
- RC3: Formant structure and variability
- RC4: Spectral bandwidth
- RC5: Voice quality and instability

### 3.2 Perceptual Analyses

The choice of simultaneous evaluation scales (Positive/Neutral/Negative and Cute/Aversive) lead to an important number of trials with a score of zero on each scale (e.g., very negative vocalizations will have zeros on the positive scale). Overall, each scale had over half of their trials scored at zero, except for Cuteness, which only had a third of zero‐scored trials. Complete score distributions for each scale are shown in Figure S1. To account for this non‐normal distribution with a positive mass at zero, the scores of each perceptual scale were entered as response variables into Generalized Linear Mixed‐Effects models with a Tweedie distribution, using the glmmTMB package (Brooks et al. 2017) in R.

Models included Valence and Age Range as interacting fixed factors, Vocalization and Participant as random intercepts, and Valence and Age Range by‐Participant slopes, as follows:

Response ∼ Age_Range * Valence

*+ (1 +Valence + Age_Range || Participant) + (1|Vocalization)*

Models were interpreted via an analysis of deviance using Type II Wald Chi‐square tests, and post‐hoc z‐tests were run using the emmeans package, compatible with glmmTMB.

In a second step, component scores from the acoustic PCA were entered into the model to assess the direct effects of acoustic parameters on participant's evaluations, as follows:

Response ∼ Age_Range * Valence + RC1 + RC2 + RC3 + RC4 + RC5

*+ (1 +Valence + Age_Range || Participant) + (1| Vocalization)*

Exploratory analyses of parentality effects were run by adding Parental Status as an interacting factor and random by‐item slope.

Response ∼ Age_Range * Valence * Parental_Status

*+ (1 +Valence + Age_Range || Participant) + (1 + Parental_Status ||Vocalization)*

## 4 Results

### 4.1 Perceptual Evaluations of Vocalizations

Summaries of the models are displayed in Table 3 and Figure 2.

**TABLE 2:** Summary of the acoustic Principal Component Analysis (adapted from Mauchand et al. (2026).

		Main parameters		
Component	Variance explained	Name	Loading	Perceptual correspondence
RC1	12.60%	F0 M (+ percentiles)	0.78	Spectral energy and pitch
		Alpha Ratio	0.89	
		Hammarberg Index	−0.78	
		Spectral slope 500–1500 Hz	0.79	
		mfcc1	−0.84	
RC2	8.30%	Loudness 20th percentile	0.76	Loudness and resonance
		Loudness sd	−0.81	
		F1 amplitude	0.75	
		F2 amplitude	0.75	
		F3 amplitude	0.74	
RC3	7.00%	F1 frequency	0.77	Formant structure and variability
		F2 frequency	0.65	
		F3 frequency	0.74	
RC4	6.00%	F2 bandwidth	0.69	Spectral bandwidth
		F3 bandwidth	0.67	
RC5	9.40%	F0 sd	0.67	Voice quality and instability
		Jitter	0.74	
		Shimmer	0.74	
		HNR	−0.83	

^*^ p < .05

^**^ p < .01

^***^ p < .001

**FIGURE 2:**
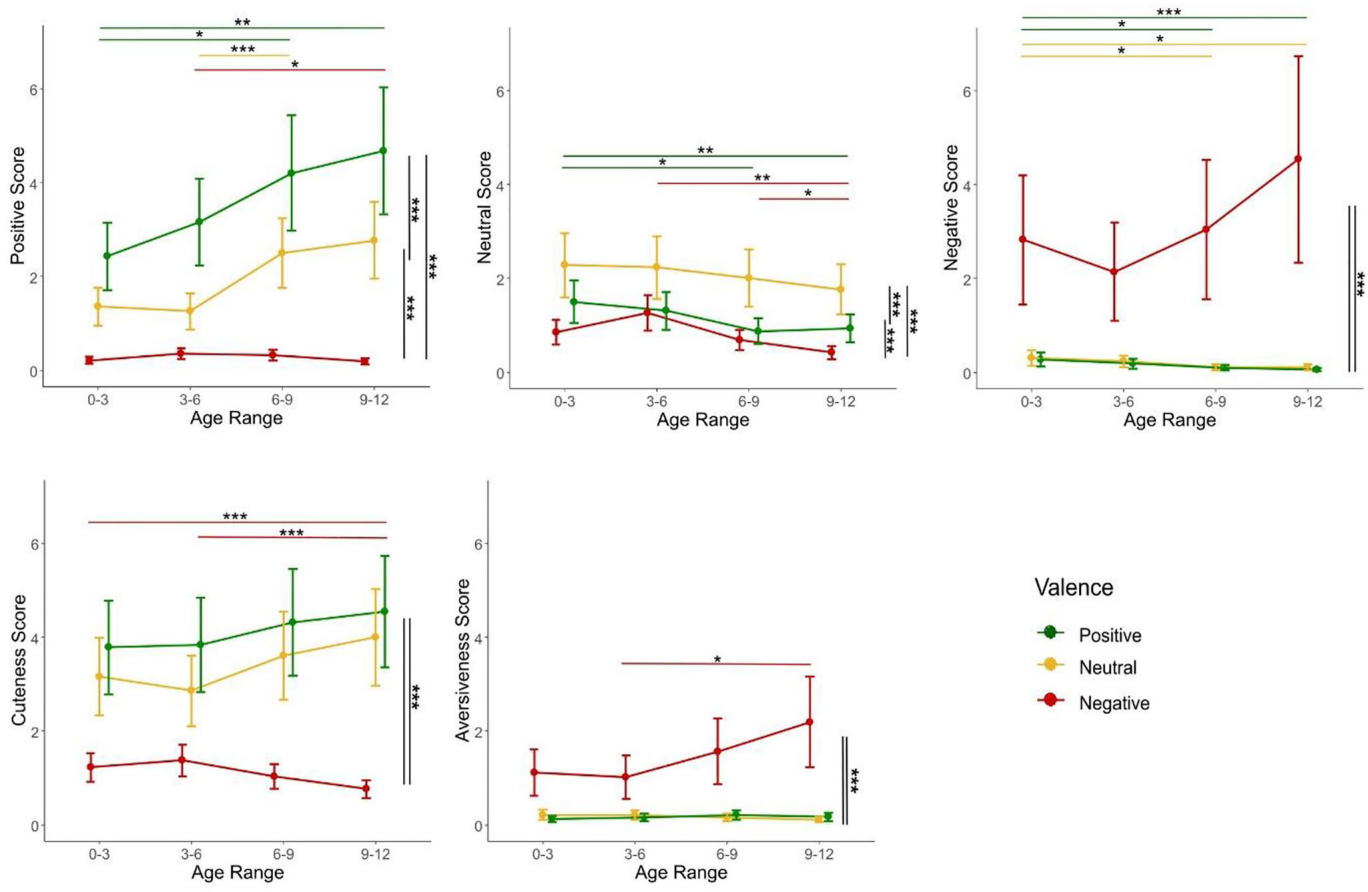
Individual vocalizations scores on each perceptual scale. Each panel represents scores from a given scale (top: Negative/Neutral/Positive, bottom: Cuteness/Aversiveness). Darker dots represent the vocalizations selected in the experimentally validated subset.

#### 4.1.1 Positivity

The evaluation of Positivity was significantly dependent on Valence (*χ* ^2^(2) = 552.57, *p* < 0.001) and Age Range (*χ* ^2^(3) = 26.35, *p* < 0.001), with a significant interaction (*χ* ^2^(6) = 23.16, *p* = 0.001). Overall, Positive vocalizations were perceived as more positive than Neutral ones (*z* = 6.18, *p* < 0.001), which were in turn more positive than Negative ones (*z* = 1.96, *p* < 0.001). As babies aged, Positive vocalizations showed a steady increase in positive ratings such that vocalizations were rated more positive at 6–9 months (*z* = 2.81, *p* = 0.026) and 9–12 months (*z* = 3.36,  .004) compared to 0–3 months. For Neutral vocalizations, a significant increase in positivity was found between 3–6 and 6–9 months (*z* = 3.40, *p* = 0.004). Negative vocalizations were rated as more positive in the 3–6 age range compared to the 9–12 age range (*z* = 2.73, *p* = 0.032).

#### 4.1.2 Neutrality

The evaluation of Neutrality was significantly dependent on Valence (*χ* ^2^(2) = 113.61, *p* < 0.001) and Age Range (*χ* ^2^(3) = 35.87, *p* < 0.001), with a significant interaction (*χ* ^2^(6) = 17.57, *p* = 0.007). Overall, Neutral vocalizations were perceived as more neutral than Positive ones (*z* = 6.33, *p* < 0.001), which were in turn perceived as more neutral than Negative ones (*z* = 4.38, *p* < 0.001). Neutral vocalizations were perceived as similarly neutral across ages (ps > 0.412). Positive vocalizations were perceived as less neutral when they were produced by babies aged 6–9 months (*z* = 3.09, *p* = 0.011) and 9–12 months (*z* = 2.72, *p* = 0.033) compared to 0–3 months. Negative vocalizations were rated as decreasingly neutral after 6 months: vocalizations were less neutral at 6–9 months than 3–6 months (*z* = 3.39, *p* = 0.004), and less neutral at 9–12 than 6–9 months (*z* = 2.64, *p* = 0.041).

#### 4.1.3 Negativity

The evaluation of Negativity was significantly dependent on Valence (*χ* ^2^(2) = 324.26, *p* < 0.001) and Age Range (*χ* ^2^(3) = 10.70, *p* = 0.013), with a significant interaction (*χ* ^2^(6) = 22.88, *p* = 0.001). Overall, Negative vocalizations were perceived as more negative than both Neutral (*z* = 14.81, *p* < 0.001) and Positive ones (*z* = 15.64, *p* < 0.001), with no significant difference between Neutral and Positive (*z* = 1.30, *p* = 0.39). Negative vocalizations were perceived as similarly negative across ages (ps > 0.128). Neutral vocalizations were perceived as less negative when they were produced by babies aged 6–9 months (*z* = –2.78, *p* = 0.028) and 9–12 months (*z* = –2.67, *p* = 0.038) compared to 0–3 months. Similarly, Positive vocalizations were perceived as less negative at 6–9months (*z* = –2.62, *p* = 0.044) and 9–12 months (*z* = –3.81, *p* = 0.001) compared to 0–3 months.

#### 4.1.4 Cuteness

The evaluation of Cuteness was significantly dependent on Valence (*χ* ^2^(2) = 343.73, *p* < 0.001), with a significant interaction with Age Range (*χ* ^2^(6) = 37.05, *p* < 0.001). Overall, Positive and Neutral vocalizations were both perceived as very cute, without significant differences between the two (*z* = 2.05, *p* = 0.101). Negative vocalizations were overall less cute than Neutral (*z* = –14.20, *p* < 0.001) and Positive (*z* = –16.08, *p* < 0.0001), with a significant decrease in cuteness at 9–12 months compared to 0–3 months (*z* = 3.72, *p* = 0.001) and 3–6 months (*z* = 4.67, *p* < 0.001). To determine the relationship between Cuteness and perceptual representations of valence, Negativity, Neutrality, and Positivity were separately entered as regressors in the Cuteness model. Positivity showed a large, positive effect (*χ* ^2^(1) = 1518.77, *p* < 0.001) which slightly reduced Valence × Age_Range effect (*χ* ^2^(6) = 27.30, *p* < 0.001), Negativity showed a large, negative effect (*χ* ^2^(1) = 551.59, *p* < 0.001) which also reduced the Valence × Age_Range effect (*χ* ^2^(6) = 20.84, *p* = 0.002), and Neutrality showed a small effect (*χ* ^2^(1) = 9.15, *p* = 0.002) which did not influence the interaction (*χ* ^2^(6) = 37.11, *p* < 0.001).

#### 4.1.5 Aversiveness

The evaluation of Aversiveness was significantly dependent on Valence (*χ* ^2^(2) = 231.49, *p* < 0.001), with a significant interaction with Age Range (*χ* ^2^(6) = 14.86, *p* = 0.021). Negative vocalizations were perceived as significantly more aversive than Neutral (*z* = 12.68, *p* < 0.001) and Positive ones (*z* = 12.59, *p* < 0.001), which did not differ (*z* = 0.27, *p* = 0.96). Aversiveness of Negative vocalizations slightly increased with age, such that they were more aversive at 9–12 months than 3–6 months (*z* = 2.69, *p* = 0.036). To determine the relationship between Aversiveness and perceptual representations of valence, Negativity, Neutrality, and Positivity were separately entered as regressors in the Aversiveness model. Negativity showed a large, positive effect (*χ* ^2^(1) = 1518.77, *p* < 0.001) which slightly reduced Valence × Age_Range effect (*χ* ^2^(6) = 27.30, *p* < 0.001), Negativity showed a large, positive effect (*χ* ^2^(1) = 666.89, *p* < 0.001) which annulled the Valence × Age Range interaction (*χ* ^2^(6) = 10.64, *p* = 0.100), Positivity showed a large, negative effect (*χ* ^2^(1) = 416.12, *p* < 0.001) but did not reduce the Valence × Age_Range effect (*χ* ^2^(6) = 13.18, *p* = 0.040), and Neutrality showed a small effect (*χ* ^2^(1) = 41.88, *p* = 0.002) which did not influence the interaction either (*χ* ^2^(6) = 14.20, *p* = 0.027).

### 4.2 Acoustic Effects on Evaluations

To assess the relationship between acoustic parameters of infant vocalizations and the perception of valence, cuteness, and aversiveness, all five PCA components were entered into the models described above. Summaries of the models are displayed in Table 3.

#### 4.2.1 Positivity

When the PCA components were entered into the Positivity evaluation model, a significant negative effect was found for RC2 (*χ* ^2^(1) = 13.25, *β* = –0.16, se = 0.04, *z* = –3.64, *p* < 0.001), suggesting that less loud and resonant vocalizations were evaluated as more positive, all else controlled for. There was also a significant negative effect of RC5 (*χ* ^2^(1) 4.43, *β* = –0.09, se = 0.04, *z* = –2.10, *p* = 0.035), suggesting that reduced noise and variability also increased evaluations of positivity. Inclusion of the PCA scores slightly reduced the main effects of Valence (*χ* ^2^(2) = 480.16, *p* < 0.001) and Age Range (*χ* ^2^(3) = 9.40, *p* = 0.024), suggesting that the acoustic parameters partly mediated these effects.

#### 4.2.2 Neutrality

All five PCA components showed significant effects when included in the Neutrality evaluation model. Positive effects were found for RC2 (*χ* ^2^(1) = 14.71, *β* = 0.13, se = 0.03, *z* = 3.84, *p* < 0.001) and RC4 (*χ* ^2^(1) = 4.75, *β* = 0.08, se = 0.04, *z* = 2.18, *p* = 0.029), suggesting that vocalizations were perceived as more neutral when they were louder, more resonant, and with larger formant bandwidths. Negative effects were found for RC1 (*χ* ^2^(1) = 97.05, *β* = –0.38, se = 0.04, *z* = –9.85, *p* < 0.001), RC3 (*χ* ^2^(1) = 6.47, *β* = –0.09, se = 0.03, *z* = –2.54, *p* = 0.011), and RC5 (*χ* ^2^(1) = 36.05, *β* = –0.19 se = 0.03, *z* = –6.00, *p* < 0.001), suggesting that vocalizations’ neutral perception was dependent on their spectral properties, reduced formants frequencies, and reduced vocal noise and variability. Inclusion of the PCA scores slightly reduced the main effect of Valence (*χ* ^2^(2) = 93.16, *p* < 0.001), suggesting that the acoustic parameters partly mediated this effect. It also cancelled the effect of Age Range (*χ* ^2^(3) = 3.13, *p* = 0.371), suggesting that the overall effect of Age Range was mostly explained by these parameters.

#### 4.2.3 Negativity

Significant positive effects were found for RC1 (*χ* ^2^(1) = 37.14, *β* = 0.49, se = 0.08, *z* = 6.27, *p* < 0.001), RC2 (*χ* ^2^(1) = 7.64, *β* = 0.20, se = 0.07, *z* = 2.78, *p* = 0.006), and RC5 (*χ* ^2^(1) = 9.65, *β* = 0.23, se = 0.07, *z* = 3.18, *p* = 0.001), suggesting that the perception of negativity was influenced by spectral and pitch properties of the vocalizations, but also by increased loudness and vocal noise and variability. Inclusion of the PCA scores slightly reduced the effect of Valence (*χ* ^2^(2) = 281.42, *p* < 0.001), suggesting that the acoustic parameters partly mediated this effect.

#### 4.2.4 Cuteness

Significant negative effects were found for RC1 (*χ* ^2^(1) = 46.12, *β* = –0.19, se = 0.03, *z* = –6.79 *p* < 0.001) and RC2 (*χ* ^2^(1) = 35.15, *β* = –0.15, se = 0.02, *z* = –5.93, *p* < 0.001), suggesting that the perception of cuteness was influenced by spectral and pitch properties of the vocalizations, as well as reduced loudness and resonance. Inclusion of the PCA scores slightly reduced the effect of Valence (*χ* ^2^(2) = 278.38, *p* < 0.001), suggesting that the acoustic parameters partly mediated this effect.

#### 4.2.5 Aversiveness

Significant positive effects were found for RC1 (*χ* ^2^(1) = 127.13, *β* = 0.70, se = 0.06, *z* = 11.28, *p* < 0.001), RC2 (*χ* ^2^(1) = 31.22, *β* = 0.31, se = 0.06, *z* = 5.59, *p* < 0.001), and RC5 (*χ* ^2^(1) = 5.88, *β* = 0.13, se = 0.05, *z* = 2.42, *p* = 0.015), suggesting that the perception of aversiveness was influenced by spectral and pitch properties of the vocalizations, as well as increased loudness, resonance, and vocal noise and variability. Inclusion of the PCA scores slightly reduced the effect of Valence (*χ* ^2^(2) = 188.27, *p* < 0.001), suggesting that the acoustic parameters partly mediated this effect.

### 4.3 Effects of Parenthood

To explore the effects of parenthood on the perception of infant vocalizations, Parental Status (Parent/Non‐parent) was entered as an interacting factor in the models (without acoustic factors). In the sample, there were 37 Parents and 42 Non‐Parents. Of note, the Parent group indexes lifetime parental experience rather than active caregiving: many of the parents were older parents who were not caring for infants at the time of participation, and current infant‐related exposure was not recorded for either group (see Limitations).

#### 4.3.1 Positivity

Parental Status significantly interacted with Valence (*χ* ^2^(2) = 7.36, *p* = 0.025). Parents gave more positive ratings than non‐parents for Negative (*z* = 2.55, *p* = 0.010) and Neutral vocalizations (*z* = 2.68, *p* = 0.008), but Positive vocalizations were perceived as equally positive by Parents and Non‐Parents (*z* = 1.37, *p* = 0.171).

#### 4.3.2 Neutrality

No significant effect of Parental Status was found.

#### 4.3.3 Negativity

Parental Status significantly interacted with Valence and Age Range (*χ* ^2^(6) = 28.73, *p* < 0.001). Overall, Parents evaluated all vocalizations as less negative than non‐parents. In the 0–3 months Range, this effect was only present for Positive vocalizations (*z* = –5.94, *p* < 0.001), but not significant for Neutral (*z* = –2.48, *p* = 0.129) or Negative vocalizations (*z* = –2.75, *p* = 0.066). In the 3–6 and 6–9 months range, the effect was present across all vocalizations (ps < 0.017). In the 9–12 months range, the effect was present for Neutral (*z* = –5.14, *p* < 0.001) and Negative (*z* = –3.02, *p* = 0.030) vocalizations, but not Positive ones (*z* = –1.34, *p* = 0.760).

#### 4.3.4 Cuteness

No significant effect of Parental Status was found.

#### 4.3.5 Aversiveness

Parental Status significantly interacted with Valence (*χ* ^2^(2) = 10.82, *p* = 0.004). Overall, Parents found all vocalizations as less aversive, although this effect was more prominent for Positive (*z* = –4.11, *p* < 0.001) and Neutral (*z* = 3.77, *p* < 0.001) than Negative vocalizations (*z* = 2.93, *p* = 0.003).

### 4.4 Evaluation Uncertainty

Participants answered “I don't know” to 275 Valence questions (2.6% of trials) and 725 Cuteness/Aversiveness questions (6.7% of trials). Uncertainty for Valence showed an effect of Parental Status (F(1, 77) = 5.96, *p* = 0.019) such that Parents showed less uncertainty than non‐parents (74 vs. 204 trials). Uncertainty for Cuteness/Aversiveness showed an effect of Valence (F(2, 336) = 11.85, *p* < 0.001) such that Positive vocalizations yielded less uncertainty (182 trials) than Negative (254 trials) and Neutral (289 trials), as well as an effect of Age Range (F(3, 336) = 16.04, *p* < 0.001), with uncertainty broadly decreasing with infant age (0–3 months: 260 trials, 3–6 months: 184 trials, 6–9 months: 156 trials, 9–12 months: 125 trials).

Participants replayed 2075 stimuli, including 205 double replays (one at each question trial). Age Range only showed an effect on replay behavior, with replays decreasing with infant age (0–3 months: 728 replays, 3–6 months: 613 replays, 6–9 months: 523 replays, 9–12 months: 416 replays).

### 4.5 Acoustic Validation of the Subset

To control for potential acoustic changes in the new subset, five ANOVA models were run for each of PCA component as the dependent variable, with Age Range, Valence, and Selection Status (selected or unselected) as interacting factors. The models revealed interacting effects of Age Range and Valence as detailed in Mauchand et al. (2026), but no effect of Selection Status except for the RC1 model, in which the effects of valence followed the same trend across stimuli (Neutral < Positive < Negative), but was more accentuated for selected stimuli (F(2, 336) = 9.29, *p* < 0.001, η^2^ = 0.05): neutral selected vocalizations had lower RC1 scores compared to unselected ones (*t*(92) = 2.22, *p* = 0.029), while negatives vocalizations had greater RC1 scores than unselected ones (*t*(89) = 4.11, *p* < 0.001). Overall, selected utterances remained acoustically representative of the whole stimuli set.

## 5 Discussion

In the present study, we aimed to develop the Geneva Infant Vocalizations of Emotion (GIVE) database: a perceptually grounded database of infant vocalizations spanning the first year of life, providing a controlled resource for future research on early emotional communication. Adult listeners’ ratings showed that vocal emotional valence is generally well recognized in infant vocalizations, even in absence of contextual information, with clear perceptual differentiation between positive, neutral, and negative categories. Building on these perceptual patterns, we derived a subset of vocalizations with the strongest alignment between experimenter‐based pre‐categorization and listener judgments, while preserving the acoustic and developmental structure of the full stimulus set.

### 5.1 Relevance and Novelty of the GIVE Database

The GIVE database currently comprises 360 high‐quality spontaneous vocalizations produced by prelinguistic infants. Vocalizations span four age ranges across the first year of development and vary in terms of expressed and perceived valence (Positive, Neutral, and Negative). For studies requiring a particularly high level of stimulus consistency (e.g., neuroimaging experiments in which the vocalizations are presented as auditory stimuli to participants), we additionally constructed a prototypical subset of 120 vocalizations. This subset was obtained by selecting stimuli for which the highest perceptual valence rating provided by listeners matched the experimenters’ initial pre‐categorization. Within each age group and valence category, the 10 most strongly and accurately perceived utterances were retained. This procedure yielded a validated stimulus set that preserves developmental variation while maximizing category clarity and perceptual distinctiveness. This subset of 120 stimuli constitutes a more manageable and reliable set than the entire database, while remaining representative of infant emotional production overall.

Several large infant vocalization corpora document early vocal development and support computational analyses of canonical versus non‐canonical vocal types, often from a cross‐cultural perspective (Cychosz et al. 2019; Ebrahimpour et al. 2020; Oller et al. 2021; Yoo et al. 2024). However, none of these datasets provide controlled, perceptually validated stimuli organized by emotional valence across age. Existing infant vocalization corpora focus specifically on the earliest pre‐babbling period (0–5 months), capturing spontaneous vocal output before canonical speech‐like structures emerge (Marschik et al. 2022), while others span broader developmental windows, from mid‐infancy to toddlerhood (Ebrahimpour et al. 2020; Pagliarini et al. 2022).

Most of these resources rely on daylong naturalistic home recordings, often collected through LENA devices, which provide high ecological validity (Li et al. 2021; Oller et al. 2021; Yoo et al. 2024). However, these databases prioritize vocal type or developmental milestones rather than listener judgments of emotional content. The GIVE database fills this gap by offering a perceptually grounded set of infant vocalizations spanning the first year of life, with documented adult judgments of emotional valence, acoustic characterization, and an experimentally derived subset optimized for future studies of infant affective signaling. Still, the database is marked by important variability across stimuli: to help future research control for this variability, each stimulus is also annotated with acoustic values for each parameter of the eGeMAPS, statistics from perceptual judgements, experimental categorization, as well as more specific details regarding infant demographics and recording context.

### 5.2 A First Look at the Database

Perceptual and acoustic results from this validation study revealed several interesting trends and patterns in infant emotional expression and their perception by adults. The main finding of these analyses is the ability of adults to infer the valence of infants’ vocalizations, even without added context and in a unimodal, auditory form. Indeed, participants largely attributed higher ratings of positivity, neutrality, and negativity to vocalizations experimentally defined as positive, neutral, and negative, respectively. These results characterize the information available in the auditory channel alone, which is precisely the condition GIVE is designed to support (e.g., neuroimaging and other auditory‐only paradigms). Unimodal recognizability sets a lower bound on, but does not describe, real‐world caregiving judgements, which integrate visual affect, context, and caregiver state; whether ratings would differ if visual information were available (e.g., an infant babbling contentedly while visibly unwell) remains to be tested.

#### 5.2.1 Perceptual‐Acoustic Markers of Infant Emotion

The present findings contribute to a growing body of work showing that adults can reliably extract broad affective information from infant vocalizations even in the absence of contextual cues. Previous studies have demonstrated that listeners almost perfectly discriminate positive from negative infant sounds, while remaining comparatively poor at identifying the specific eliciting situation (i.e., the specific context or cause that produced the vocalization, such as hunger, pain, being left alone, or play) (Lindová et al. 2015). Our own annotation scheme records comparable eliciting contexts (parental scolding, a loud noise, a comfort episode, a crying phase, play, or the absence of a parent or of stimulation), but these served to define the experimenters’ pre‐categorization of valence rather than to be identified by listeners. Similarly, validation work on the Oxford Vocal Sounds (OxVoc) database confirms robust adult categorization of infant cries and laughs by valence, although neutral infant vocalizations often remain perceptually ambiguous (Young et al. 2016). Extending this literature, our results provide novel evidence that adults’ perception of infant vocal valence is not static across infancy but sharpens as infants develop across the first year: positive and neutral vocalizations are judged increasingly positive with age, whereas negative sounds become more clearly negative after mid‐infancy. This developmental trajectory likely reflects systematic changes in the acoustic form of infant vocalizations (Mauchand et al. 2026), consistent with prior continuum models showing that perceived distress is graded and predicted by loudness, pitch, spectral concentration, and vocal instability across cries and speech‐like vocants (Yoo et al. 2019). Our multidimensional acoustic analyses go beyond these distress‐focused accounts: the same acoustic components (RC1, spectral energy and pitch; RC2, loudness and resonance; RC5, voice quality and instability) predicted several of the perceptual scales, with opposite signs for positivity and cuteness on the one hand and for negativity and aversiveness on the other (Table ^3^), pointing to partly shared acoustic bases for these evaluative dimensions. How the acoustic dimensions themselves change across development is analyzed in a companion paper (Mauchand et al. 2026).

**TABLE 3:** Summary of statistical models (GLMMs with Tweedie distribution) for each perceptual scale. The left columns describe the models which did not included acoustic parameters, while the right columns show the full models including acoustics (as well as the direction of each acoustic effect).

Model Dependent variable	Factor	No acoustic parameters			With acoustic parameters			
		*χ* ^2^	df	*p*	*χ* ^2^	df	*p*	Direction of acoustic effect
Positivity	Age_Range	26.35	3	<0.001***	9.40	3	0.024*	
	Valence	552.57	2	<0.001***	480.16	2	<0.001***	
	Age_Range:Valence	23.16	6	<0.001***	24.66	6	<0.001***	
	RC1				0.73	1	0.394	
	RC2				13.25	1	<0.001***	—
	RC3				0.72	1	0.395	
	RC4				3.05	1	0.081	
	RC5				4.43	1	0.035*	—
Neutrality	Age_Range	35.87	3	<0.001***	3.13	3	0.372	
	Valence	113.61	2	<0.001***	93.16	2	<0.001***	
	Age_Range:Valence	17.57	6	0.007**	15.29	6	0.018*	
	RC1				97.05	1	<0.001***	—
	RC2				14.71	1	<0.001***	+
	RC3				6.47	1	0.011*	—
	RC4				4.75	1	0.029*	+
	RC5				36.05	1	<0.001***	—
Negativity	Age_Range	10.70	3	0.013*	11.96	3	0.008**	
	Valence	324.26	2	<0.001***	281.42	2	<0.001***	
	Age_Range:Valence	22.88	6	<0.001***	28.55	6	<0.001***	
	RC1				37.14	1	<0.001***	+
	RC2				7.64	1	0.006**	+
	RC3				1.23	1	0.267	
	RC4				2.60	1	0.107	
	RC5				9.65	1	0.002**	+
Cuteness	Age_Range	0.39	3	0.942	2.35	3	0.503	
	Valence	343.73	2	<0.001***	278.38	2	<0.001***	
	Age_Range:Valence	37.05	6	<0.001***	43.87	6	<0.001***	
	RC1				46.12	1	<0.001***	—
	RC2				35.15	1	<0.001***	—
	RC3				1.77	1	0.184	
	RC4				1.03	1	0.310	
	RC5				1.87	1	0.172	
Aversiveness	Age_Range	1.61	3	0.658	4.17	3	0.243	
	Valence	231.49	2	<0.001***	188.27	2	<0.001***	
	Age_Range:Valence	14.86	6	0.021*	15.81	6	0.015*	
	RC1				127.13	1	<0.001***	+
	RC2				31.22	1	<0.001***	+
	RC3				0.05	1	0.816	
	RC4				1.00	1	0.318	
	RC5				5.88	1	0.015*	+

The acoustic–perceptual links observed in the present paper converge with prior evidence on how adults decode distress and aversiveness in infant vocalizations, while adding nuance about when specific cues become clearly negative. The present results show that loud, resonant vocalizations are perceived as more negative and less positive, consistent with findings that distress‐related infant vocalizations exhibit higher intensity and spectral energy than positive ones (Mauchand et al. 2026; Yoo et al. 2019), and with classic work linking sound pressure level and high‐frequency energy to aversiveness (Gustafson and Green 1989). Importantly, loudness alone also increased neutrality ratings, suggesting that intensity may signal (non‐)positivity rather than a linear index of valence. The present study further identifies instability and noise as robust markers of negativity and aversiveness, in line with continuum models of graded distress (Yoo et al. 2019) and evidence that nonlinear, harsh vocal phenomena bias listeners toward more negative interpretations (Anikin 2020).

The present findings are consistent with evidence that acoustically complex and irregular vocal patterns, such as nonlinear phenomena or roughness, bias listeners toward perceiving more negative and high‐arousal affect, whereas simpler, softer, and more regular sounds are more often interpreted as neutral or positive (Anikin 2020; Liuni et al. 2020). This aligns with work showing that infant vocalizations with smoother spectral profiles elicit more positive evaluations, while increased pitch, intensity, and instability contribute to heightened negativity and aversiveness (Arteche et al. 2024). Elevated pitch and high‐frequency energy similarly increased perceived negativity, supporting accounts that these cues reflect arousal and vocal effort (Mauchand et al. 2026). Because loudness and pitch cues are also tied to the infant's arousal state (Wass et al. 2020), and arousal was not rated in the present study, perceived valence and arousal cannot be disentangled here. Together, these findings support a “vocal tension” framework in which negative affect is most strongly inferred from the combination of loudness, spectral sharpness, and instability (McKenna and Stepp 2018), whereas formant‐related parameters appear to modulate perceived neutrality or typicality rather than valence itself (Arteche et al. 2023; Mauchand et al. 2026). Squeals and growls constitute an informative boundary case for this account: they can be loud and spectrally extreme, which would predict aversiveness, yet playful squeals and growls are plausibly heard as cute or affiliative, suggesting that the mapping between acoustic tension and perceived valence is not strictly monotonic. Because vocalization type was not annotated in GIVE, we cannot currently locate such vocalizations within the perceptual space; adding vocalization‐type annotations is a priority for the next release of the database.

#### 5.2.2 Cuteness, Aversiveness, and Caregiving Signals

Adult perception of infant vocalizations is shaped not only by broad emotional valence but also by spontaneous evaluative dimensions of cuteness and aversiveness, which play complementary roles in caregiving‐related responses. Research on auditory cuteness demonstrates that even neutral, vowel‐like infant sounds elicit a specific positive implicit association compared with adult or animal vocalizations, suggesting that infant voices carry an intrinsic affiliative bias beyond simple valence coding (Arteche et al. 2023, 2024). Our results confirm this suggestion, as Neutral and Positive vocalizations alike elicited high ratings of cuteness, with little differences across development. Cuteness may here be a “default” perceptual property of infant vocalizations—a stable, auditory baby schema (Kawaguchi and Waller 2024; Lorenz 1943) which would only be dissipated by strong negative, distress cues. This is further confirmed by the effects of perceptual valence ratings on cuteness: contrary to aversiveness effects, which were fully driven by perception of negativity, cuteness effects were affected by valence ratings of both negativity and positivity—and only in a partial manner. The stability in the perceptual representation of cuteness is also strengthened by the absence of parenthood effects: parents and non‐parents were both very sensitive to cuteness, as detailed further below. Emotional expression of positivity thus develops in a relatively decorrelated way from external evaluative characteristics of cuteness; these findings nuance and extend recent auditory baby‐schema accounts emphasizing that positive infant sounds such as laughter and babbling rapidly capture attention and promote playful, approach‐oriented caregiving (Kringelbach et al. 2016; Xu et al. 2019). Cuteness appears here as a stable, default characteristic of infant behavior, and a potentially universal perceptual construct targeting investment from *any* adult.

At the same time, infant cries are strongly evaluated along an aversive and urgent dimension: longer duration, high fundamental frequency, dysphonation, and concentrated high‐frequency energy reliably increase perceived unpleasantness and distress (Gustafson and Green 1989; Yoo et al. 2019). Work on nonlinear vocal phenomena further shows that harsh, chaotic voice quality systematically biases listeners toward more negative and aversive interpretations, highlighting the importance of acoustic instability as a cue to urgency (Anikin 2020). Although both parents and non‐parents are highly sensitive to distress acoustically (Lindová et al. 2015; Yoo et al. 2019), caregiving experience modulates evaluative responses: parents and especially mothers often report stronger positive engagement with infant sounds and show enhanced neural responsiveness in reward and emotion circuits (Kringelbach et al. 2016; Parsons et al. 2017). Overall, infant vocalizations are heard through a dual perceptual lens, in which soft, stable, high‐pitched sounds are coded as cute and affiliative, whereas loud, noisy, and spectrally extreme cries are coded as aversive and urgent, reflecting an adaptive system that supports both nurturant and protective caregiving behavior (Parsons et al. 2014).

#### 5.2.3 Emotion Development and Functional Flexibility

Developmentally, the period around 6 to 9 months appears as a critical milestone after which the emotional expressivity of infants significantly progresses: negative vocalizations become increasingly negative, positive (and even neutral) vocalizations become increasingly positive, and neutrality decreases overall. This 6–9 months milestone has been previously reported in a variety of other aspects of vocal and emotional development (Kent and Murray 1982; Lieberman 2018; Snow 2006). The present perceptual findings also corroborate previous acoustic classification models showing improved accuracy for vocalizations over 6 months (Mauchand et al. 2026), suggesting that accurate representation of infant emotions by adults relies on the infant's developing abilities to acoustically encode these emotions. On the other hand, this seemingly contrasts with accounts of “functional flexibility” of infants’ vocal productions: Oller et al. (2013), in particular, report that most vocalizations are “protophones” whose acoustic features do not consistently encode specific emotional states; in such cases, emotion attribution is performed by adults based on other facial or contextual cues and the intersubjective emotional experience between adults and infants. Our findings suggest instead that emotional valence is at least partly encoded (and decoded) in the vocalization signal, independent of surrounding context, especially after 6 months. Still, the present data may be reconciled with Oller's accounts in the light of the progressive perceptual confounding of Neutral and Positive vocalizations after 6 months. Here, the neutral protophones seem to exhibit a form of functional flexibility, and in a low‐stakes context like the experiment, be attributed to a more positive valence.

#### 5.2.4 Parenthood

Finally, our results speak to experience‐dependent modulation of infant sound perception: while earlier work reports modest effects of parenthood on emotional resonance and processing of infant cues (Lindová et al. 2015; Mizuguchi et al. 2024; Parsons et al. 2017), we find that parents tend to evaluate neutral and negative sounds more positively. This pattern admits two readings: parents may show a softening of negative appraisal or, conversely, non‐parents may show heightened distress sensitivity or a tendency to interpret ambiguous sounds more negatively, for instance, if they are less comfortable with infants. Our between‐groups design cannot distinguish between these accounts, and disentangling them would require a measure of baseline affinity or comfort with infants. Both readings are compatible with evidence that infant cries exert disproportionate cognitive impact and attentional capture in adults (Dudek et al. 2016), as well as neuroimaging work showing altered salience and affective processing of infant vocalizations in caregivers (Parsons et al. 2017; Rigo et al. 2025). Taken together, our study advances current debates from whether adults can detect infant affect to how listeners’ judgements of valence, aversiveness, and cuteness change with infant age and caregiver experience; the acoustic trajectory of these vocalizations across the first year is analyzed in a companion paper (Mauchand et al. 2026), whereas here acoustics enter only as trial‐level predictors of perception. Of note, however, our Parent group indexes lifetime parental experience rather than active caregiving: many of the parents studied were older parents who were not actively caring for infants, and current infant‐related exposure was not recorded in either group. More broadly, parenthood is a coarse proxy for infant‐related experience and motivation. Parental status was the only listener‐level variable collected, and baseline affinity toward infants may modulate ratings of cuteness and aversiveness independently of parenthood (and might likewise shape any auditory baby‐schema effect). Future data collection with GIVE should include such measures.

#### 5.2.5 Operationalization of Valence and Construct Validity

A central caveat concerns how valence is operationalized in GIVE. Valence is defined here by the convergence of two adult‐based judgements: an expert, context‐informed pre‐categorization (which relies on caregiver responses and interactional context, but not on visual infant affect) and independent ratings by naive listeners. No independent measure of the infant's internal state (e.g., facial affect or physiology) was available. GIVE therefore validates the perceptual recognizability of contextually defined valence categories, not the ground truth of infant affect: some vocalizations labeled positive may not have co‐occurred with positive infant affect, and some labeled negative may not have reflected distress. For the same reason, we refer throughout to perceived valence, cuteness, and aversiveness; arousal was not rated, and our conclusions are limited to acoustic correlates of listeners’ perceptual judgements rather than to reliable acoustic markers of the infants’ emotional state. Synchronized video and physiological measures are the natural validation route for future corpora.

### 5.3 Limitations

Despite the strengths of the GIVE database, several limitations should be acknowledged. First, the stimulus set is characterized by substantial natural variability across vocalizations, reflecting differences in infants’ developmental stage, recording environments, and eliciting contexts. While this ecological richness is a key feature of the corpus, it may also introduce uncontrolled acoustic and situational heterogeneity that could influence perceptual judgments. In particular, the corpus spans two sets of recordings, albeit with comparable audio qualities. To support future work in controlling for this variability, each stimulus is therefore accompanied by detailed annotations, including acoustic descriptors from the eGeMAPS parameter set, summary statistics from perceptual ratings, experimental categorizations, and information on infant demographics and recording context. Second, although the present analyses identify robust perceptual dimensions of valence, cuteness, and aversiveness, further investigations with larger samples and complementary neuroimaging approaches will be necessary to test the neural and developmental mechanisms underlying these effects.

Moreover, the valence labels of GIVE rest on audio‐only recordings. Both corpora were recorded without video, and the expert pre‐categorization was based on the vocalization and its auditory context alone. The labels thus index adult‐inferred valence in context rather than independently verified infant affect, and may not always reflect the infant's actual emotional state. It is important to note that the initial valence pre‐categorization, based on acoustic contextual cues surrounding each vocalization, was intended to balance the corpus rather than establish ground‐truth emotional labels; accordingly, the subsequent perceptual evaluation by adult listeners constituted the main validation step for the database.

Furthermore, vocalization type was not annotated in the current release of the database. The variables recorded during segmentation were infant age, age range, sex, vocalization duration, and expert‐assigned valence. Consequently, we cannot quantify the representation of specific vocalization types (e.g., babbles, squeals, or growls) or examine how the relationship between perceived valence and vocalization type changes across development. This limits our ability to directly address accounts emphasizing the functional flexibility of infant vocalizations. In addition, we did not collect qualitative feedback on participants’ experience of the perceptual task, such as whether evaluating valence, cuteness, and aversiveness from auditory‐only stimuli was easy, difficult, ambiguous, or fatiguing. Such feedback could have provided further context for interpreting the ratings; the proportion of “I don't know” responses therefore remains the only available indirect indicator of uncertainty or difficulty in making these judgments.

As for parental responses, parental status reflected lifetime parental experience rather than current caregiving. Neither current exposure to infants nor participants’ affinity toward infants was assessed. Accordingly, differences associated with parental status should be interpreted cautiously and considered exploratory. Rather than representing a primary aim of the study, these analyses illustrate one of the potential applications of GIVE for investigating how individual experience may relate to adults’ responses to infant vocalizations.

Finally, the size and structure of the dataset may limit some prospective analyses. The relevant constraint is not only the number of stimuli but also their nesting: the validated subset comprises 120 vocalizations across 12 age × valence cells, drawn from 30 infants (35 for the full set of 360 vocalizations; Table S1), some of whom contribute vocalizations at more than one age range, which restricts designs requiring infant‐level random effects. The fixed selection rule (the 10 best “correct” vocalizations per cell) was chosen to keep the subset balanced by design. A concordance‐threshold rule would have yielded more stimuli but unbalanced cells: as Table ^1^ shows, the number of correctly categorized vocalizations ranges from 28/30 (e.g., Positive, 6–9 months) to 10/30 (Neutral, 9–12 months), so a threshold would leave the neutral cells at older ages thinly populated, precisely where the perceptual ambiguity of interest lies. Users can nevertheless apply their own threshold to the full 360‐stimulus set, which is released with its perceptual statistics.

### 5.4 Conclusions, Further Research and Use of the Stimuli

With GIVE, we aimed to provide a validated, comprehensive, and manageable database of vocal stimuli that capture a wide range of infant's expressive abilities across the early stages (pre‐linguistic) of development. Our first analyses on the database confirmed the acoustic and perceptual relevance of the stimuli and began to unveil the developmental trajectories of the perception of emotional expression. They revealed acoustic correlates of listeners’ perceptual judgements of valence from early stages of emotional expression, a perceived refining of the emotion repertoire after 6 months, as well as their relationships with second‐hand perception of cuteness, aversiveness by adults, including caregivers. These results characterize the information available in the auditory channel alone and should not be generalized to multimodal caregiving contexts.

We aim to further develop and populate this database with infant stimuli from other cultures and languages, to assess environmental factors in emotion development. As it stands, the GIVE database constitutes relevant stimuli set for future studies on infant communication, caregiving behavior, parentality. In particular, the validated subset can be used as a reliable set for studies requiring a high level of stimulus control, such as neuroimaging experiments, in which the vocalizations are presented as auditory stimuli to participants. Together with our preliminary analysis and annotations, this subset will also allow future researchers to investigate more complex representations of the vocalizations while relying on previous acoustic and perceptual evidence.

## Author Contributions

**Maël Mauchand**: Conceptualization, Investigation, Writing – original draft, writing – review and editing, Data curation, Supervision, Formal analysis. **Irene Lorenzini**: Data curation, Writing – original draft, writing – review and editing, Resources, Funding acquisition. **M. Gratier**: data curation, resources, writing – review and editing. **L. Pugin**: data curation, investigation, writing – review and editing. **A. Gomes–fernandes**: investigation, data curation, writing – review and editing. **L. Ceravolo**: writing – review and editing, validation. **D. Grandjean**: funding acquisition, supervision, writing – review and editing. **M. Filippa**: conceptualization, methodology, writing – original draft, writing – review and editing, validation.

## Funding

Open access funding provided by the University of Geneva. This work was supported by the Swiss National Science Foundation (SNSF) through the NCCR Evolving Language (Agreement no. 51NF40_180888), and by the MSCA Individual Fellowship INCODE to I.L. (grant agreement no. 895484).

## Ethics Statement

The study was validated by the University of Geneva Ethics Board (CUREG‐MM‐2023‐12‐166).

## Conflicts of Interest

The authors declare no conflicts of interest.

## Supporting information




**Supporting information**: desc70304‐sup‐0001‐SuppMat.docx

## Data Availability

The data that support the findings of this study, as well as sample vocalizations, are openly available in OSF at https://osf.io/nd894, reference number nd894. The database generated during the current study will be made available upon request to the Swiss Center for Affective Sciences (CISA), University of Geneva, at https://www.unige.ch/cisa/research/materials‐and‐online‐research/research‐material, with access granted for academic research purposes upon reasonable request.
