# Supplementary material for: The GIVE Database: Acoustic and Perceptual Characteristics of Infant Emotional Vocalizations in the First Year of Life
Source: Dev Sci. 2026 Nov 11;30(1):e70304. doi: 10.1111/desc.70304 (PMC13755160; doi:10.1111/desc.70304)
Supplement: Supplementary file 1 [file DESC-30-e70304-s001.docx]

**SUPPLEMENTARY MATERIALS**

**Table S1.** Evolution of vocalization count across segmentation and selection progess. * 0-3 and 3-6 age ranges share 4 speakers.

| Age Range | Original Corpus | Total Segmented | | Curated (part of GIVE) | | Validated (part of subset) | |
| --- | --- | --- | --- | --- | --- | --- | --- |
|  |  | N vocs | N infants | N vocs | N infants | N vocs | N infants |
| 0-3 | EVOC | 162 | 10 | 90 | 10 | 30 | 9 |
| 3-6 | EVOC | 250 | 9 | 90 | 8 | 30 | 7 |
| 6-9 | INCODE | 158 | 13 | 90 | 13 | 30 | 10 |
| 9-12 | INCODE | 125 | 8 | 90 | 8 | 30 | 8 |
| TOTAL | | 695 | 36* | 360 | 35* | 120 | 30* |
|  |  |  |  |  |  |  |  |
|  | | | |  |  |  |  |

**Figure S1.** Distribution density of responses to each perceptual scale.
